# Characterization of Indoor Air Quality on a College Campus: A Pilot Study

**DOI:** 10.3390/ijerph16152721

**Published:** 2019-07-30

**Authors:** Grant Erlandson, Sheryl Magzamen, Ellison Carter, Julia L. Sharp, Stephen J. Reynolds, Joshua W. Schaeffer

**Affiliations:** 1 Department of Environmental and Radiological Health Sciences, Colorado State University, Fort Collins, CO 80523, USA; 2 Department of Civil and Environmental Engineering, Colorado State University, Fort Collins, CO 80523, USA; 3 Department of Statistics, Colorado State University, Fort Collins, CO 80523, USA

**Keywords:** indoor air, sustainability, LEED, higher education, public health, particulate matter

## Abstract

Recent construction trends on college campuses have demonstrated a shift to designing buildings with features focused on sustainability. However, few studies have investigated indoor air quality in institutions of higher education, particularly in sustainably designed buildings. The objective of this study was to evaluate the association of building and occupancy on indoor air quality within and between higher education buildings. We measured particulate matter, formaldehyde, carbon dioxide, and nitrogen oxides in LEED certified, retrofitted, and conventional building types on a college campus. Three size fractions of particulate matter were measured in each building. We conducted multi-zonal, 48-h measurements when the buildings were occupied and unoccupied. Outdoor particulate matter was significantly higher (PM2.5 = 4.76, PM4 = 17.1, and PM100 = 21.6 µg/m^3^) than in classrooms (PM2.5 = 1.7, PM4 = 4.2, and PM100 = 6.7 µg/m^3^) and common areas (PM2.5 = 1.3, PM4 = 4.2, and PM100 = 4.8 µg/m^3^; all *p* < 0.001). Additionally, concentrations of carbon dioxide and particulate matter were significantly higher (*p* < 0.05) during occupied sampling. The results suggest that occupancy status and building zone are major predictors of indoor air quality in campus buildings, which can, in turn, increase the concentration of contaminants, potentially impacting occupant health and performance. More research is warranted to reveal building features and human behaviors contributing to indoor exposures.

## 1. Introduction

The United States Environmental Protection Agency and the National Human Activity Pattern Survey estimate that the average American spends approximately 90% of their time indoors [1,2,3]. Occupants of indoor environments, including homes, workplaces and schools are exposed to a mixture of pollutants with known health effects, including volatile organic compounds (VOCs) [4,5], particulate matter (PM) [6,7,8,9,10,11,12], nitrogen dioxide [13], allergens and other biological exposures [14,15]. The contribution and concentration of these pollutants are influenced by various sources, including outdoor air, occupant behavior, building materials, building practices and operations (e.g., air exchange rates) [16]. Consequently, health effects among occupants can range from fatigue, headache, and eye irritation to more serious outcomes such as shortness of breath, allergic responses and asthma exacerbations [8,9,17,18,19,20,21,22,23,24,25,26]. Particular attention has been given to PM exposure and the associated health effects in recent studies [7,8,9,10,11,12]. The 2009 EPA Integrated Science Assessment for Particulate Matter identified a causal relationship between PM_2.5_ exposure and respiratory inflammation, allergic responses, and cardiovascular effects ranging from increased heart rate to mortality [8]. VOCs, specifically formaldehyde in indoor air have also been of increasing concern and have been the focus of past studies [25,27,28,29,30,31]. A recent controlled exposure study established subjective sensory irritation thresholds for short-term exposure to formaldehyde; IARC has recognized formaldehyde as a carcinogen in chronic exposures [28,29]. In addition to adverse respiratory and somatic symptoms, poor indoor air quality (IAQ) can have a direct and indirect impact on occupant performance of cognitive-based tasks [21,22,23,24,32,33]. In their experimental study, Satish et al. found a decrease in computer-based decision making tests when participants were exposed to elevated levels of CO_2_ [22]. Additional observational studies conducted in K-12 schools indicated decreased performance on computer, numerical, and language-based tasks in elevated CO_2_ environments [21,23,24]. Indirectly, poor IAQ in classrooms can lead to increased absenteeism and to decreased academic performance [34].

The higher education sector represents a unique environment in that it acts as a work environment for faculty, a learning environment for students, and frequently, a home environment for students. Each year in the United States, an estimated 20 million students enroll at nearly 4,600 college level institutions staffed by 4 million faculty and employees [35,36]. Given the magnitude of the population impacted, there is a need to characterize and understand indoor environmental quality in higher education buildings. Compared to K-12 school settings, research focused on IAQ in higher education buildings has been limited, with most studies largely focusing on environmental quality factors and perception-based measures [32,33,37,38]. Very few studies have investigated exposure to particulate matter and attendant health impacts in the university setting [37,39]. Moreover, additional research is needed to understand distributions of particulate matter within and between university buildings, especially in the context of green building construction.

Currently, little information detailing the inventory or conditions of college campus buildings exists, but construction data provide some insight. For example, construction spending on new projects has more than tripled that of renovations and additions to existing buildings, suggesting a shift toward projects that involve new construction [40]. This has led to a drop in allocation of funds to repair existing buildings and HVAC systems, leading to poorly managed ventilation rates, one of the primary determinants of IAQ [40]. New construction on college and university campuses reflect a nationwide shift toward design and the construction of high-efficiency and low emitting buildings, many of which adhere to various green building standards. The most widely used green building certification in the world is LEED (Leadership in Energy and Environmental Design), with an estimated 92,000 projects comprising 19.3 billion square feet worldwide [41].

A recent study investigating air quality between LEED buildings and conventional buildings indicated better perceived IAQ in LEED certified buildings [42]. Additional studies have explored the role of indoor environmental quality and its role in occupant perception and satisfaction [43,44]. However, no research has investigated quantitative IAQ measurements between building types. Further, the characterization of IAQ within and between different higher education building types has yet to be explored. The objective of this research was to quantify objective measures of IAQ and evaluate whether these measures vary within building zones, by building type, and by occupancy status. Here, we report novel data on size-segregated PM, aldehydes, and CO_2_ concentrations in traditional, retrofitted, and newly constructed “green” (or LEED certified) buildings on the campus of a large, public university. From an intra-building perspective, we hypothesized that outdoor building zones would have the highest concentrations of contaminants, followed by the common area building zones and classroom building zones. We also hypothesized that the newly constructed LEED certified building would exhibit the lowest level of contaminants, followed by the retrofitted building, and conventional building. Finally, we hypothesized that all building types and zones would have higher contaminant concentrations when occupied compared to when they were unoccupied. By testing these hypotheses, we gained insights into the contaminants present in a campus setting and the role intra- and inter-building differences play in IAQ, and more broadly the implications of new building practices on IAQ.

## 2. Materials and Methods

### 2.1. Sample Collection

**Figure 1:**
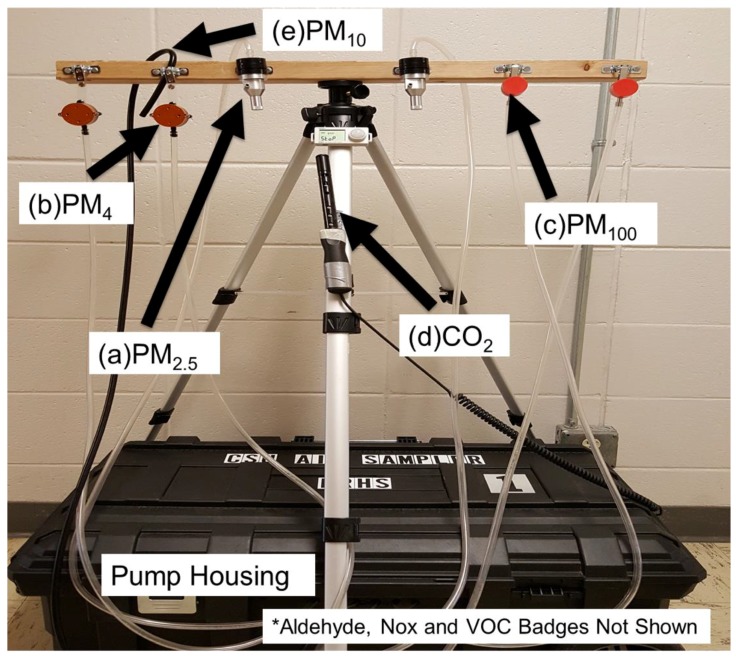
Customized air sampling station deployed in multiple test locations to characterize indoor air quality in different building types on a college campus. The design of this station allowed for easy integration of (**a**) two GK2.05 (KTL) aluminum cyclones for collecting PM_2.5_; (**b**) two aluminum parallel particle impactors for collecting PM_4_; (**c**) two SKC Button samplers for collecting PM_100_; and (**d**) one TSI Q-trak for monitoring CO_2_ in real-time. Passive badges (not shown) for measuring NO_x_, aldehydes, and VOCs were affixed to the horizontal bar during sample collection. SKC sampling pumps were housed inside the station (black box), which contained a fan for climate control.

Sampling was conducted at a public research university in the Mountain West, which is characterized by a semi-arid high desert climate with an average annual temperature of 10 °C. This region receives 16 in of yearly rainfall and 57 in of yearly snowfall [45]. The study site included 705 buildings across five campuses and services approximately 33,800 students and over 6000 employees. Three educational buildings were selected to span the range of construction age and building design on campus; a newly constructed LEED-gold building, a retrofitted building, and a conventional building. Newly constructed buildings were defined as recently constructed in the last 10 years and built to LEED certified gold status for new construction. LEED gold certification status is the third highest of four certification levels requiring 60–79 credits out of a possible 100 [46]. Retrofitted buildings were defined as conventional buildings that had received updates to the building air handling systems and building materials in the last ten years. Conventional buildings were defined as those that had not been updated within the past 20 years and were not constructed with any green building certifications. Additionally, each building had to contain at least two active classrooms, one common area, and a secure outside location. Of the buildings that met the building type criteria, those in proximity to our research laboratory were selected for convenience of transportation and deployment of air sampling stations (Figure 1).

Indoor air samples and environmental conditions were collected during six distinct sampling campaigns across three seasons (Fall, Winter, Spring) over a year long period. In each season, one 48-h sampling campaign was conducted while the building was occupied and one while it was unoccupied. Th occupied samples were collected during normal building and classroom operations. The unoccupied samples were collected during campus shutdowns such as breaks and holidays. In each sampling campaign, air sampling stations were deployed in the following four building zones: two classrooms, one common area, and one outside. The common area zone was selected to represent an area typical of heavy traffic/activity between and during classes. Classroom zones were determined by selecting room sizes commonly found in university buildings that were used primarily for lectures. The outdoor zone was selected to provide a measure of ambient pollutant levels directly outside the building.

Samples were collected using customized air sampling stations consisting of a pump/instrument housing and an elevated platform (approximately 1.5 m off the ground) for co-located active and passive samplers (Figure 1). Air sampling stations were deployed in fixed building zones within each building. Classroom stations were placed at the front of the room, common area stations along the wall of a high traffic area, and outdoor stations positioned near the entrance/exit of the building. Sample collection periods spanned 48-h to account for potential day-to-day variability. The outdoor samples were collected over one eight-hour period during the second day of sampling due to concerns regarding weather and security.

### 2.2. Air Quality Measures

*Particulate Matter.* Each sampling station included a total of six samplers (including replicates) for measuring PM_100_, PM_4_, and PM_2.5_ using two SKC Buttons (SKC Inc., Eighty-four, PA, USA), two SKC aluminum parallel particle impactors (PPI), and two BGI GK2.05 (KTL) aluminum cyclones (Mesa Labs, Lakewood, CO, USA). All samplers were loaded with 5 µm pore size polyvinyl chloride filters and calibrated to the desired flowrate of 4 liters per minute (L/min). Flow calibration was performed before and after each campaign using a primary flow standard and differences of less than 5% were considered acceptable.

*Aldehydes.* Each sampling station was equipped with UMEx 100 passive samplers (SKC, Inc., Eighty-Four, PA, USA), which were used to measure formaldehyde and acetaldehyde concentrations. These samplers were positioned above the other samplers with the inlet pointed away from the wall to limit the effect of airflow produced by adjacent active samplers. During the fall and winter sampling campaigns, a sampler was deployed at each sampling location to collect a 48-h sample. Outdoor sampling stations were outfitted with one UMEx sampler to collect an 8-h sample. Locations of the badges on sampling stations remained constant throughout all sampling campaigns. 

*Nitrogen oxides.* An Ogawa passive sampler (Ogawa USA, Pompano Beach, FL, USA) was affixed to each air sampling station. Samplers were loaded with 14.5 mm triethanolamine pre-coated filters to capture the following compounds: NO_x_, NO, and NO_2_. Filters were stored at freezing temperatures (<0 °C) pre- and post-sampling until analysis was performed. Ogawa samplers were included only during the winter and spring campaigns due to sampler availability issues. The position of the Ogawa sampler (as with the Umex 100 sampler) remained constant across all campaigns. One Ogawa sampler was used at each sampling location inside and outside each building for the 48-h and 8-h sampling periods, respectively.

*Environmental Measurements.* The Q-Trak (7575, TSI, Inc., Shoreview, MN, USA) was deployed to characterize (in real-time) environmental conditions, including carbon monoxide, carbon dioxide, temperature, and relative humidity measurements. The Q-Trak instruments logged data at 1-minute intervals over the 48-h and 8-h sampling periods. The Q-Trak probe was extended outside the pump housing and positioned near the PM samplers.

### 2.3. Sample Analysis

#### 2.3.1. Gravimetric

The total mass of each size fraction of PM collected was determined by weighing a filter using an anti-static system and a microbalance balance (Mettler-Toledo, Inc., Columbus, OH, USA). Filter samples were desiccated for a minimum of 24 h before weighing (pre- and post-sampling). Filters were weighed in duplicate, and the average of two measurements was used to compute the difference between pre- and post-sampling mass of each filter. Airborne concentrations were calculated by dividing the blank-corrected gross mass of PM on each filter by the volume of air sampled. Laboratory and field filter blanks were used to correct for measurement error and background. Filter samples were subsequently archived at −80 °C for future analyses to determine the biological and chemical composition of the different aerosol fractions.

#### 2.3.2. High Performance Liquid Chromatography

Analysis of UMEx 100 samplers was performed in accordance to the EPA IP-6C method as previously described [47]. Samples were extracted by desorbing the filter in 3 mL of acetonitrile and aliquoting 120 µL of sample into HPLC autosampler vials. Calibration standards were produced by reconstituting DNPH-formaldehyde and DNPH-acetaldehyde powder (AccuStandard, New Haven, CT, USA) with acetonitrile. Two HPLCs were utilized interchangeably throughout sampling campaigns; a Waters 2690 separations module and an Agilent 1290 Infinity. To maintain consistency across both instruments, the chromatography column (Zorbax Eclipse Plus C18) was transferred between instruments and sample run parameters (including UV detection at 365 nm) were identical. Filter and solvent blanks were included in each analysis and served as reference and control for background contamination.

#### 2.3.3. Spectrophotometry

Analysis of NO_x_ compounds was completed through spectrophotometry on a BioTek Synergy HTX multi-mode reader by following the Ogawa specific NO, NO_2_, NO_x_ and SO_2_ sample analysis protocol [48]. Samples were extracted with 8 mL of HPLC grade water and mixed with 2 mL of color producing reagent. Standards were created by diluting a previously prepared nitrite stock solution. Standards and samples were aliquoted into a 96 well plate and analyzed at a wavelength of 545 nm. Filter and assay reagent blanks were included for reference and control. NO_x_ concentrations were calculated according to a standard curve using Gen5 software. A limit of detection (LOD) for this study was determined to be 9 ppb and limit of quantitation (LOQ) of 30 ppb based on empirical data and the NIOSH Manual of Analytical Methods (NMAM).

### 2.4. Statistical Analysis

Data were organized based on building type, occupancy status, building zone, and analyte. Geometric means and geometric standard deviation were reported for all constituents for consistency with reporting of log-transformed measurements. Data were then log transformed to test for a log normal distribution typically found in air quality measurements. Comparisons of log concentrations between occupancy status and zone within buildings were conducted using a linear mixed effects model with occupancy, zone, and the occupancy by zone interaction as fixed effects and building type as a random block effect. Follow-up comparisons using Fisher’s Least Significant Difference were conducted to examine relevant differences between building zones and occupancy status when a significant effect was observed in the mixed effects model. Model assumptions were verified using residual plots. Because only one building of each type was used in the study, descriptive statistics were computed and compared qualitatively. All statistical analyses were conducted with R software (R 3.4.0, R foundation for statistical computing: Vienna, Austria). A significance level of 0.05 was used for all tests of significance.

## 3. Results and Discussion

A total of 341 samples were collected in three different building types across six sampling campaigns during three sampling points over a one-year period. Indoor and outdoor samples were collected over 48 h and 8 h, respectively. The average weekly occupancy by classroom is presented in Table 1.

Descriptive statistics for PM_100_, PM_4_, PM_2.5_, formaldehyde, carbon dioxide, and nitrogen oxide samples are presented in Table 2. 

The PM geometric mean concentrations were 7.46 (SD = 2.42) µg/m^3^, 5.35 (SD = 2.27) µg/m^3^, and 1.95 (SD = 2.23) µg/m^3^ for PM_100_, PM_4_, and PM_2.5_, respectively. The average concentrations of formaldehyde and CO_2_ were 6.91 µg/m^3^ (*n* = 39) and 493 ppm (*n* = 61). Each CO_2_ sample represents an average of one-minute readings taken during the sampling period.

### 3.1. Comparisons by Building Zone and Occupancy

#### 3.1.1. PM

Least squares mean average log concentrations of particulate matter are presented by building zone and occupancy status in Table 3 below.

**Figure 2:**
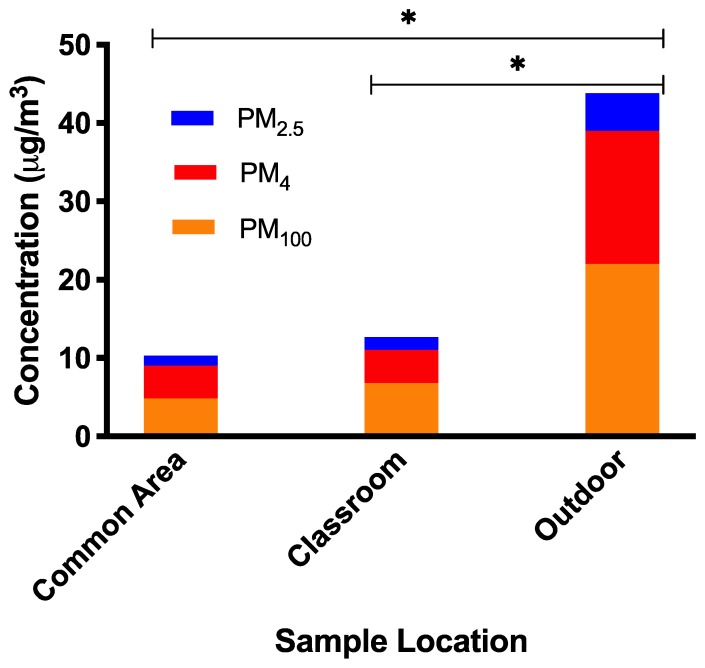
Average PM concentrations by size-fraction observed in each sample location. Stacked bar graph of unoccupied and occupied PM_100_, PM_4_, and PM_2.5_ concentrations by sample location. Outdoor concentrations at PM_100_ (bottom bar), PM_4_ (middle bar), and PM_2.5_ (top bar) were significantly higher (* signifies significance at *p*-value < 0.05) than concentrations in the other two sample locations.

The three PM size fractions had similar patterns of average log concentrations at the three zones, although the interaction between occupancy status and zone within buildings was significant for only PM_4_ and PM_100_ (*p* = 0.030 and *p* = 0.0061, respectively; Figures S1–S3). For all PM in common areas and classrooms, the PM was higher for occupied spaces than for unoccupied spaces, although this difference was only significant for PM_100_ classrooms (*p* = 0.0151). For outdoor zones, samples taken while the building was occupied were lower than while it was unoccupied, but these differences were not significant for any PM. Exterior spaces had average log concentrations of PM_4_ and PM_100_ that were significantly higher than the common areas and classrooms in unoccupied spaces (all *p* < 0.001), but unoccupied common areas and classrooms did not significantly differ (PM_4_ *p* = 0.9935 and PM_100_ *p* = 0.9390). There were not significant differences in PM_4_ or PM_100_ between the occupied zones (all *p* > 0.05). The average log PM_2.5_ significantly differed by zone within building, with outdoor spaces having significantly higher concentrations than the common areas and classrooms, regardless of occupancy status (both *p* < 0.001). Geometric mean concentrations by building zone are presented in Figure 2.

High outdoor PM levels relative to indoor concentrations are to be expected due to a lack of major indoor combustion sources within the building, maintained ventilation systems, and low occupant activity levels (primarily students seated in class). There were higher levels of PM in classrooms as compared to common areas, although the difference was not significant; the higher classroom PM levels was unexpected. Common areas have a higher activity level than classrooms (walking vs sitting) and should have a higher infiltration rate, as common areas were directly connected to entrances. A possible explanation is that classrooms are heavily occupied for a larger percentage of the day than common areas. For example, aside from periodic passing periods the number of occupants in the common areas was generally low while classrooms were occupied and in session. We hypothesize that the level of occupancy has a greater impact (leading to higher concentrations in classrooms) than the types of activities undertaken in the common area between classes. It is important to note that while we found significant differences in concentrations of particulate matter, none of our samples approached ambient or occupational exposure limits. Thus, the practical implications of these observed differences are unknown and warrant further investigation as it relates to characterizing and contextualizing PM in campus indoor air.

#### 3.1.2. Formaldehyde

The formaldehyde concentrations measured in the unoccupied common areas (3.96 µg/m^3^ (SE = 0.152) and the occupied common areas (3.86, SE = 0.131) were similar to those measured in the unoccupied and occupied classrooms (3.88, SE = 0.112 and 3.74, SE = 0.0993, respectively). There was no significant interaction effect between occupancy status and building zone on log formaldehyde concentration (*p* = 0.887; Figure S4), nor were there significant zone (*p* = 0.425) or occupancy status main effects (*p* = 0.3124). It is possible that the classrooms sampled were constructed with lower emitting materials, leading to lowered levels, but without additional information about the specific materials utilized in construction, little can be inferred from the result. Indoor formaldehyde concentrations are primarily produced by furniture, wood products, and building material emissions. Therefore, occupancy is not expected to have a large impact on concentrations. Outdoor formaldehyde measurements were excluded from analysis due to outdoor sampling constraints.

#### 3.1.3. CO_2_

There was no significant interaction effect between zone and occupancy status for average CO2 (Figure S5). Average CO_2_ levels were highest in classrooms (513 ppm), common areas demonstrated the second highest concentrations (498 ppm), and outdoor areas had the lowest levels (425 ppm) among test zones within buildings, although there was no significant zone main effect. Using CO_2_ as an indicator for occupancy, it is understandable that classrooms that are occupied much of the day have higher levels than common areas that are only full during passing periods. This helps explain the increase in CO_2_ in classrooms when compared to common areas. There was a significant occupancy status main effect, with occupied spaces having higher average log CO2 (603 ppm) than unoccupied spaces (423 ppm), regardless of building zone (*p* < 0.001).

Significant differences in CO_2_ between occupancy groups were expected because in buildings without a large combustion source, humans are the primary contributor to increased levels of carbon dioxide [49]. Moreover, levels of carbon dioxide during unoccupied conditions in all three building types and zone types were near the ambient conditions (402 ppm) referenced by the National Oceanic and Atmospheric Administrations, which is anticipated in empty buildings [50].

### 3.2. Comparisons Between Buildings

#### 3.2.1. PM

**Figure 3:**
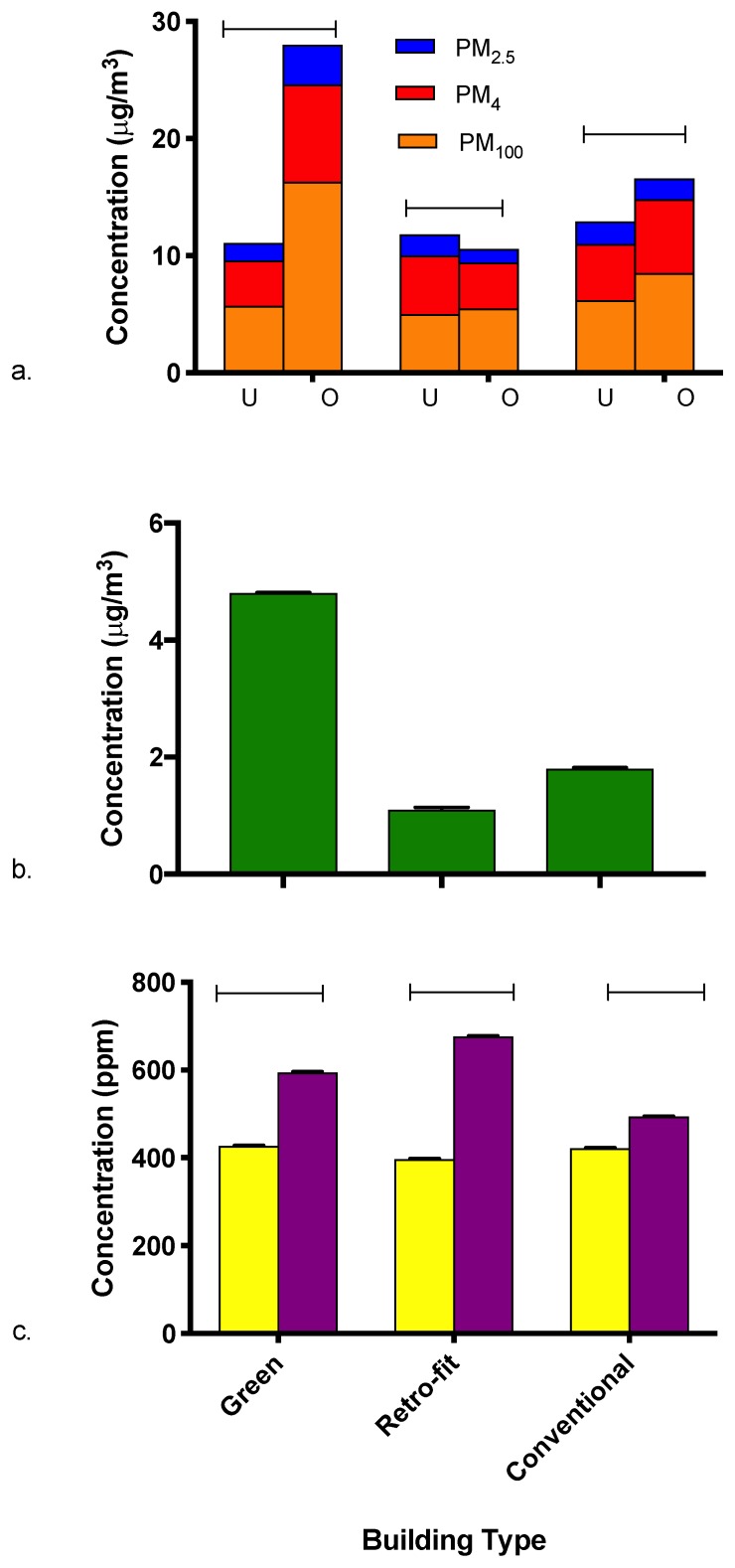
Indoor air quality measures by building type and occupancy: (**a**) airborne concentrations of PM_100_, PM_4_, and PM_2.5_ by building and occupancy; (**b**) formaldehyde concentrations by building; and (**c**) CO_2_ concentrations by building and occupancy. Because formaldehyde concentrations were not statistically significant between occupied and unoccupied, only an aggregated value by building is presented. Occupied status is represented as (U) unoccupied (left bar) and (O) occupied (right bar) in panels a and c.

PM_100_ concentration differences were observed across the different building types. The retrofitted building type had the lowest average concentration of PM_100_ (5.2 µg/m^3^ (2.46)), followed by the conventional building type (7.3 µg/m^3^ (1.93)), and the newly constructed LEED building type (9.4 µg/m^3^ (2.73)). PM_2.5_ concentrations also showed variation among building types. PM_2.5_ was lowest in the retrofit building 1.5 (2.14) µg/m^3^, while the conventional and LEED buildings exhibited higher concentrations of 1.9 (2.06) µg/m^3^ and 2.3 (2.44) µg/m^3^. On the contrary, PM_4_ concentrations did not substantially vary by building type with average concentrations of 4.43 (2.25) µg/m^3^, 5.51 (2.13) µg/m^3^, and 5.63 (2.34) µg/m^3^ for the retrofit, conventional, and LEED building. PM concentrations by building type are presented in (Figure 3a).

We hypothesized that all concentrations in the LEED building would be the lowest. Instead, data for two of the three size fractions (PM_100_ and PM_2.5_) indicate that the newly constructed green building had higher concentrations than the other two buildings. These findings may be attributed to (1) longer durations spent occupied (Table 1), and/or (2) interior negative pressure within the building. During sampling periods in the LEED certified building, we observed vents/registers that had been modified by occupants (e.g., cardboard affixed to the vent to block/redirect air flow). While we could not confirm that similar occupant actions weren’t also taken in the retrofitted and conventional buildings, activities such as these could have disrupted how building air flows were managed and led to elevated indoor PM levels. Furthermore, blocking vents can generate a negative pressure environment, which limits the ability of the building envelope to keep outdoor PM out. Higher occupancy in the LEED-certified building may also have provided greater opportunity for resuspension of particulate matter from carpeting and surfaces [51].

#### 3.2.2. Formaldehyde

Formaldehyde concentrations were highest in the newly constructed green building (4.8 µg/m^3^), followed by the conventional building (1.8 µg/m^3^), and the retrofit building (1.1 µg/m^3^) (Figure 3b). These concentrations are comparable to indoor formaldehyde concentrations reported in previous research [27,29,52]. Additionally, observed concentrations fall well below the World Health Organization guideline and the American Conference of Governmental Industrial Hygienists threshold limit value for occupational exposures.

The average concentrations between buildings do not support the initial hypothesis of lower formaldehyde levels in the LEED building. The LEED certified building was built with low emitting building materials and would be expected to have lower levels than the other buildings that didn’t follow LEED requirements. However, emission decay was not considered when the hypothesis was proposed. A typical source of formaldehyde indoors is building materials, and the emission source strength of these materials is anticipated to decline with time, resulting in lower indoor formaldehyde levels. A study on particleboard, a building material containing formaldehyde, found an average half-life of 216 days across 16 products [53]. Considering the age of the conventional building type, the formaldehyde available for emission from the building materials used during construction is likely to have been substantially depleted over time. Conversely, the retrofitted and newly constructed green buildings have had much less time to off gas VOCs present in their building materials.

#### 3.2.3. CO_2_

The newly constructed green building had the highest mean CO_2_ concentrations (518 ppm), followed by the retrofitted building (512 ppm), and the conventional building (455 ppm). Slightly higher CO_2_ concentrations were noted in the LEED and retrofitted buildings, which are likely related to higher occupancy observed in those buildings when compared to the conventional building (Figure 3c). At the building level, the increase in CO_2_ among all three buildings while occupied can be explained by the level of occupancy each building experienced under the assumption that occupants are the main source of indoor CO_2_. The conventional building was observed with a small number of occupants during a given sampling period and therefore would be expected to have a smaller difference between occupancy statuses. A larger number of occupants was observed in both the retrofitted and green buildings, leading to a greater difference between occupancy statuses. Concentrations differences seen in the green building could also be partly attributed to occupants tampering with the ventilation during occupied sampling. Blocking of air vents observed in the LEED building could have limited air flow concentrating CO_2_ within the building.

#### 3.2.4. Nitrogen Oxides (NO_x_)

A total of 70 samples were analyzed for NO_x_ using spectrophotometry with an LOQ of 30 ppb. Only one sample (36 ppb) was above the LOQ. Additionally, only 13 of 70 samples exceeded the limit of detection (9 ppb). Due to this limitation, no statistical analyses were performed on either nitrogen dioxide or nitrogen monoxide. The expected indoor NO_2_ concentrations were around the limit of quantitation for our equipment [25,54]. The concentrations in this study were also expected to be low, as no major NO_x_ sources (e.g., cooking operations) were present in these non-residential indoor settings [55,56]. While no indoor NO_x_ limit or guideline has been established, the average annual and one-hour NO_2_ guidelines set by the WHO is 40 µg/m^3^(~21 ppb) and 200 µg/m^3^(~106ppb) respectively. Our highest measured 48-h NO_2_ concentration was 14 ppb, which was well below both guideline values.

### 3.3. Limitations

Our study was primarily limited by a small number of buildings sampled out of the 705 possible buildings at the study site. This small sample of buildings limits the generalizability of this study to other campus buildings and, more broadly, to all higher education buildings. However, intra- and inter-building differences that we observed suggest directions for future work and underscore the value of measuring multiple constituents, locations, and time points. Sample size, along with heterogeneity of weather within the seasons, also limited our ability to utilize season as a block in statistical analysis. Therefore, potential seasonal variations in fan operations were not addressed in our analysis. Future studies should utilize a larger sample size within season and longer sampling periods to obtain homogeneity within seasons. This study was also limited because we did not report air pollutant concentration averages separately for daytime and nighttime sampling periods, in part due to resource and instrumentation limitations, as well as the risk of collecting insufficient mass with shorter sampling times, which could be a priority to overcome in future studies. However, we note that the periods classified as occupied samples included nighttime periods when the buildings were occupied to a much lesser extent. Because of the paucity of research on this IAQ topic (i.e., indoor air quality in higher education campus buildings), this study provides valuable insight for future studies and attendant a priori determinations despite low study power.

## 4. Conclusions

Individuals spend significant time indoors, particularly as an increasing proportion of the United States population lives in urbanized settings. Therefore, it is essential to understand the indoor environment and potential harmful exposures found within it. Inhalation is a major route of exposure to environmental pollutants indoors, and indoor environmental exposures have been associated with various negative health outcomes. Characterizations of indoor air pollutants and their subsequent health outcomes have been conducted in elementary schools and office settings. However, the unique characterization of indoor air quality at American institutes of higher education has been almost entirely absent from the literature. Recent transitions to more efficient and sustainable campus buildings, alongside long durations of student exposures, further motivate the need to quantify indoor air quality in higher education. Using common campus buildings, we characterized indoor air quality across three different higher education building types. Three size fractions of particulate matter, formaldehyde, carbon dioxide, and nitrogen oxides were compared within and between building type as well as across occupancy statuses. Building zone had a significant impact on indoor air quality levels. The outdoor measurements for nearly all measured analyses were higher than in indoor common areas or classrooms, as we had hypothesized. To a lesser extent, building type also influenced air quality levels. However, LEED certification was found to have no positive impact on air quality, contrary to our initial hypothesis. Instead, our results suggest that while being built with efficiency and sustainability in mind, air quality measurements were higher in the LEED-certified building type. Among both building type and zone, occupied samples exhibited higher concentrations than unoccupied samples, which was consistent with our hypothesis. Based on these findings, PM_100_ should be included in future IAQ investigations (which typically focus on smaller size fractions of particulate matter), as these particles can deposit in the upper and lower respiratory system and lead to both acute and chronic adverse health effects. This study provides an important reference and starting point for future research into indoor air and indoor environmental quality in campus facilities at institutes of higher education. More broadly, this study adds to the growing evidence base for understanding the implications of recent shifts in building construction toward features aimed at sustainability and energy efficiency associated with a “green” building environment.

## Figures and Tables

**Table 1:** Average time occupied per week by classroom and semester.

Building/Classroom	Spring Semester Weekly Occupancy (hours)	Fall Semester Weekly Occupancy (hours)
Traditional/Room 1	6.0	8.0
Traditional/Room 2	13.0	16.0
Retrofitted/Room 1	29.1	26.6
Retrofitted/Room 2	31.9	36.9
LEED Building/Room 1	29.6	35.7
LEED Building/Room 2	29.6	31.6

**Table 2:** Average concentrations of air quality measures across independent sampling campaigns in each building.

Analyte	*n*	Min	Geometric Mean (SD)	Max
CO_2_ (ppm)	61	271	493 (1.34)	986
Formaldehyde (µg/m^3^)	39	0.75	6.91 (2.32)	34.25
PM_100_ (µg/m^3^)	67	1.10	7.46 (2.42)	52.75
PM_4_ (µg/m^3^)	69	1.25	5.35 (2.27)	45.4
PM_2.5_ (µg/m^3^)	70	0.55	1.95 (2.23)	11.15
NO_x_ (ppb)	35	<LOQ	<LOQ	36.31

<LOQ: below the limit of quantitation.

**Table 3:** Least squares mean average log concentrations of each size fraction of particulate matter by occupancy status and zone within buildings in µg/m^3^.

Analyte	Occupancy Status	Common Area	Classroom	Outdoor
PM100	Occupied	0.756 (0.125)	0.978 (0.106)	1.234 (0.161)
	Not Occupied	0.591 (0.125)	0.691 (0.106)	1.613 (0.149
PM4	Occupied	0.714 (0.092)	0.763 (0.081)	1.137 (0.137)
	Not Occupied	0.486 (0.094)	0.538 (0.081)	1.378 (0.116)
PM2.5	Occupied	0.175 (0.113)	0.316 (0.086)	0.644 (0.159)
	Not Occupied	0.092 (0.113)	0.184 (0.087)	0.780 (0.133)

## Supplementary Materials

The following are available online at https://www.mdpi.com/1660-4601/16/15/2721/s1, Figure S1: PM_4_ occupancy status and building zone interaction plot (*p* < 0.030), Figure S2: PM_2.5_ occupancy status and building zone interaction plot, Figure S3: PM_100_ occupancy status and building zone interaction plot (*p* < 0.0061), Figure S4: Formaldehyde occupancy status and building zone interaction plot, Figure S5: CO_2_ occupancy status and building zone interaction plot.
