# Supplementary material for: Characterization of Indoor Air Quality on a College Campus: A Pilot Study
Source: Int J Environ Res Public Health. 2019 Jul 30;16(15):2721. doi: 10.3390/ijerph16152721 (PMC6695958; doi:10.3390/ijerph16152721)
Supplement: Supplementary file 1 [file ijerph-16-02721-s001.zip › ijerph-549488-supplementary.pdf]

Supplemental Materials *for*

Characterization of Indoor Air Quality on a College Campus: A Pilot Study

Grant Erlandson [1], Sheryl Magzamen [1], Ellison Carter [2], Julia L. Sharp [3], Stephen J. Reynolds [1] and Joshua W. Schaeffer [1,*]

[1] Department of Environmental and Radiological Health Sciences, Colorado State University, Fort Collins, CO 80523, USA; erlandso@colostate.edu (G.E.); Sheryl.Magzamen@colostate.edu (S.M.); Stephen.Reynolds@colostate.edu (S.J.R.)

[2] Department of Civil and Environmental Engineering, Colorado State University, Fort Collins, CO 80523, USA; Ellison.Carter@colostate.edu

[3] Department of Statistics, Colorado State University, Fort Collins, CO 80523, USA; Julia.Sharp@colostate.edu

* Correspondence: Joshua.Schaeffer@colostate.edu; Tel.: +1-970-491-6636

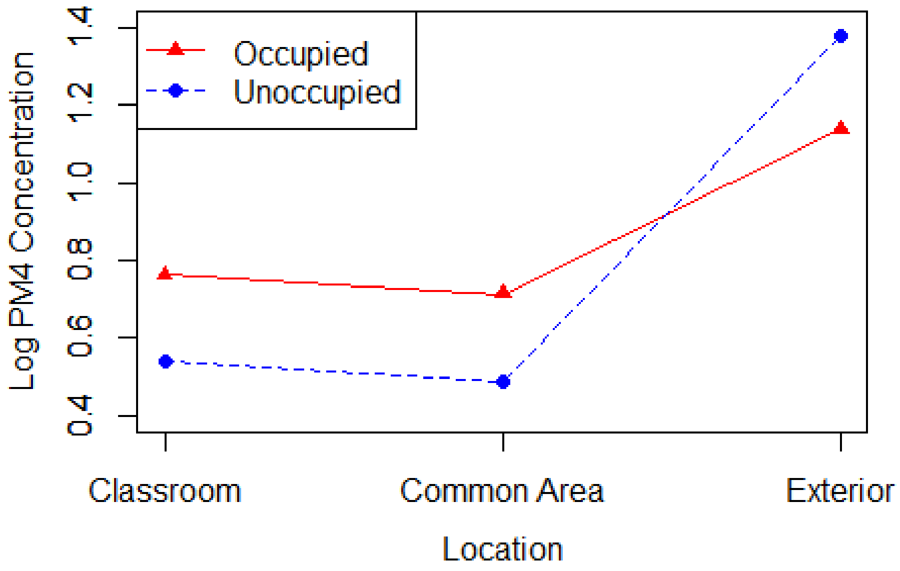

Figure S1. PM_4 occupancy status and building zone interaction plot ($p < 0.030$).

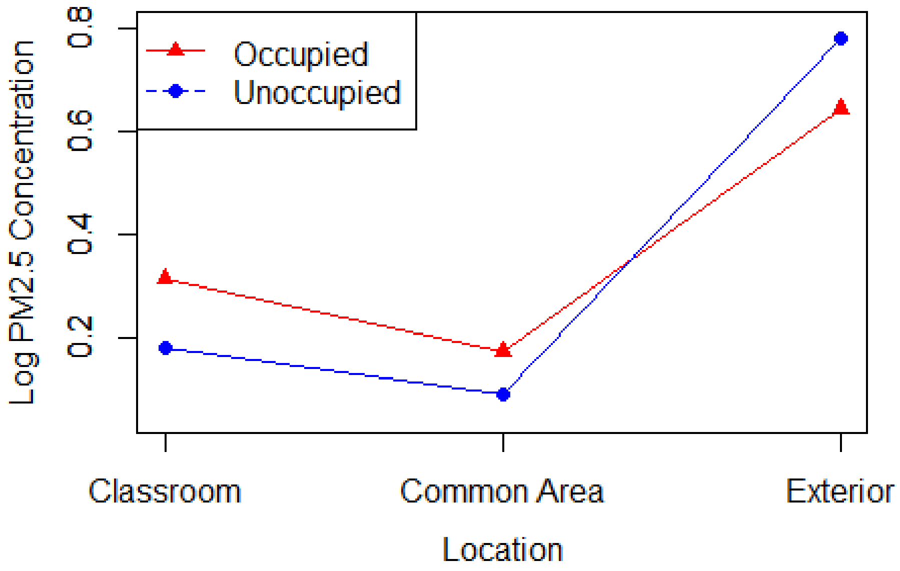

Figure S2. $PM_{2.5}$ occupancy status and building zone interaction plot.

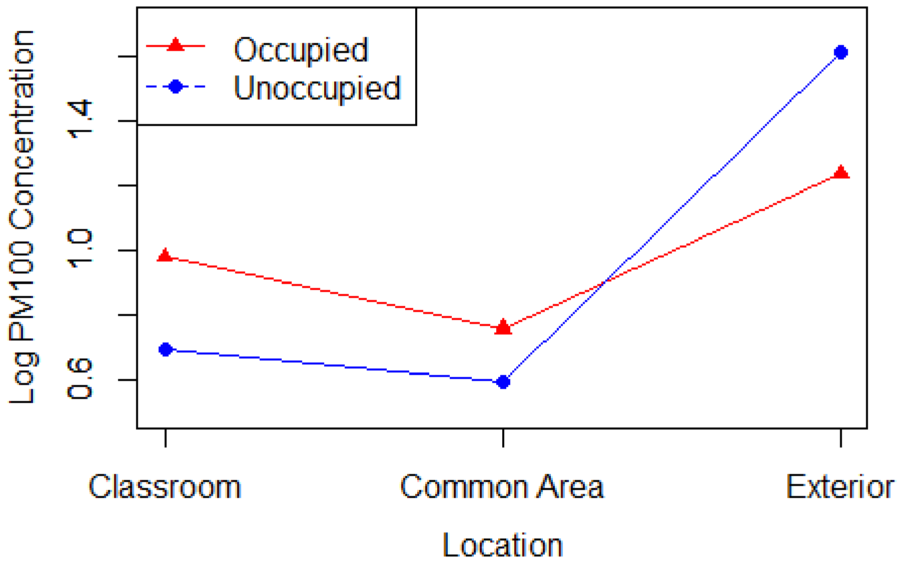

Figure S3. PM_{100} occupancy status and building zone interaction plot ($p < 0.0061$).

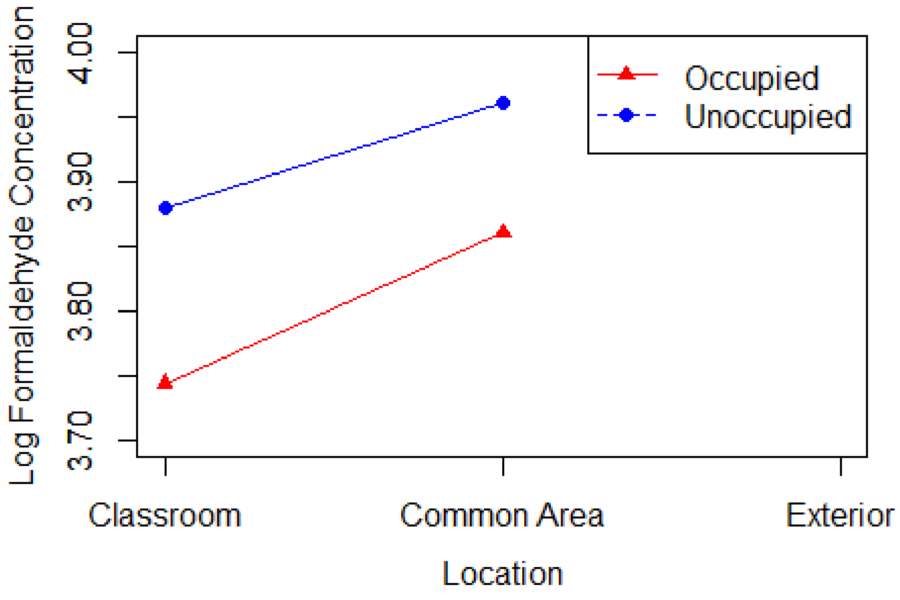

Figure S4. Formaldehyde occupancy status and building zone interaction plot.

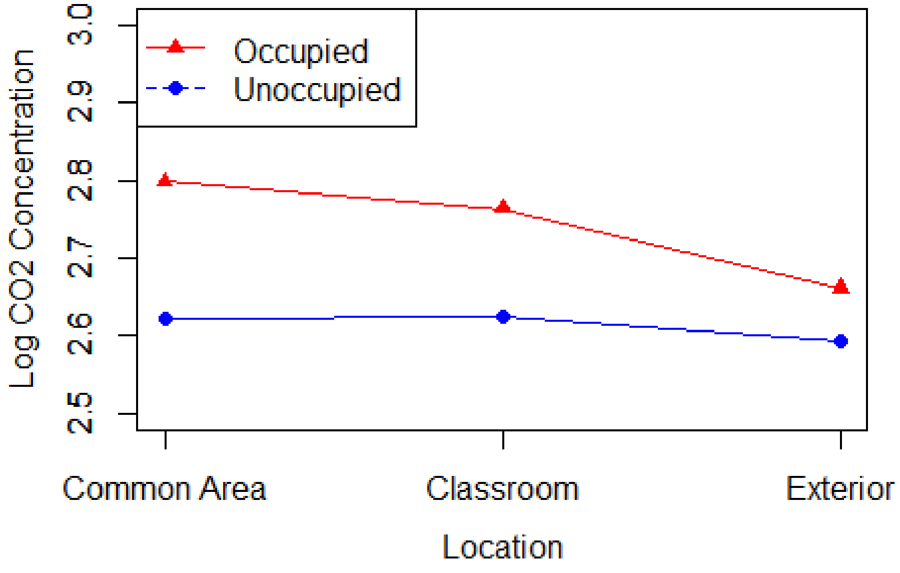

Figure S5. CO_2 occupancy status and building zone interaction plot.
